# Insulin secretion pattern in patients with congenital adrenal hyperplasia during the hyperglycemic clamp compared with a control group

**DOI:** 10.1186/1758-5996-7-S1-A110

**Published:** 2015-11-11

**Authors:** Daniel Minutti de Oliveira, Ezequiel Moura Gonçalves, Renata Isa Santoro de Pieri, Daniella Fernandes Camilo, Ana Carolina Vasques, Sofia Helena Lemos Marini, Gil Guerra, Bruno Geloneze Neto

**Affiliations:** 1 UNICAMP, Campinas, Brazil

## Background

Intrauterine hypocortisolism and postnatal corticosteroids chronic replacement, which does not mimic the physiological pattern, are characteristic of patients with the classic form of congenital adrenal hyperplasia (CAH) by deficiency of the 21-hydroxylase enzyme (21OHD). Long-term effects studies of this treatment on insulin secretion pattern by pancreatic beta cells are scarce.

## Objectives

Evaluate the function of pancreatic β cells and peripheral insulin resistance through the hyperglycemic clamp in patients with the classic form of CAH-21OHD and compare with healthy individuals.

## Materials and methods

The hyperglycemic clamp was applied, the gold standard method for evaluating secretory function of pancreatic beta cells.

## Results

The patients with CAH-21OHD, showed an increase of insulin resistance and compensatory insulin secretion resulting in normal glucose homeostasis.

## Conclusions

Young adult patients with CAH-21OHD have a normal glucose metabolism by functional adaptation of hypersecretory beta cell. Strategies for the maintenance of the physiological adaptive pattern should be developed to ensure long-term normoglycemia in this specific population.

